# Road Topology Refinement via a Multi-Conditional Generative Adversarial Network

**DOI:** 10.3390/s19051162

**Published:** 2019-03-07

**Authors:** Yang Zhang, Xiang Li, Qianyu Zhang

**Affiliations:** 1 School of Electronic Science, National University of Defense Technology (NUDT), Changsha 410073, China; lixiang01@vip.sina.com; 2 School of Business, University of Leeds, Leeds LS2 9JT, UK; zhang7yu@126.com

**Keywords:** multi-conditional generative adversarial network, road topology refinement, road network extraction

## Abstract

With the rapid development of intelligent transportation, there comes huge demands for high-precision road network maps. However, due to the complex road spectral performance, it is very challenging to extract road networks with complete topologies. Based on the topological networks produced by previous road extraction methods, in this paper, we propose a Multi-conditional Generative Adversarial Network (McGAN) to obtain complete road networks by refining the imperfect road topology. The proposed McGAN, which is composed of two discriminators and a generator, takes both original remote sensing image and the initial road network produced by existing road extraction methods as input. The first discriminator employs the original spectral information to instruct the reconstruction, and the other discriminator aims to refine the road network topology. Such a structure makes the generator capable of receiving both spectral and topological information of the road region, thus producing more complete road networks compared with the initial road network. Three different datasets were used to compare McGan with several recent approaches, which showed that the proposed method significantly improved the precision and recall of the road networks, and also worked well for those road regions where previous methods could hardly obtain complete structures.

## 1. Introduction

**Figure 1:**
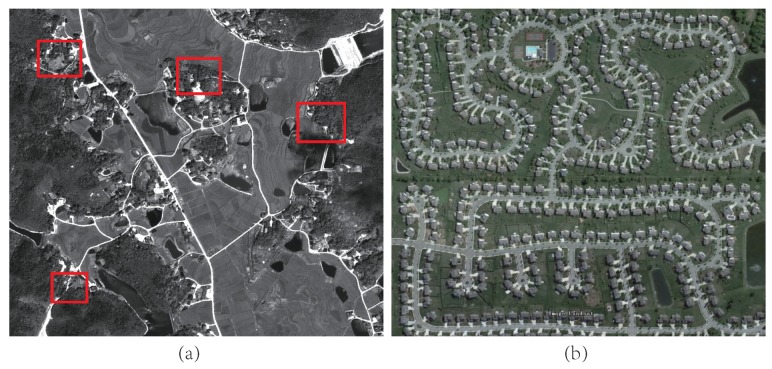
Comparison of the road: in rural region (**a**); and in urban region (**b**). The red boxes in (**a**) indicate some abnormal spectral road regions, where are challenging for road extraction.

Road topology reconstruction is a fundamental yet long-standing problem for remote sensing applications [1,2,3], thus receiving wide attention in the past decades. Complete road topological networks are widely used in many fields, such as traffic flow monitoring [4], self-driving technology [5], intelligent public transportation [6], navigation [7], road map construction [8], traffic incident detection [9,10], etc. However, most methods cannot produce satisfactory road networks, due to the complex spectral condition of road area. For example, as shown in Figure 1a, the road situation could be very complex in the rural region. The width, materials and surrounding environment of rural roads are diverse, which lead to varying spectral performance in the image. Such characteristics make it more challenging to reconstruct complete road topological networks for the rural area; for example, disconnection and distortion often appear in extracted road networks [3,11].

To get more complete and correct road networks, although previous road extraction methods have achieved great progress, there still exists some inherent drawbacks. Most of the feature-based approaches rely on the spectral behavior or intensity contrast [12], thus relying heavily on appropriate features to describe the road regions [3,13]. This kind of methods may be limited by the various spectral behaviors from different satellites [14]. To address these issues, recent works [1,2,3,15] try to reconstruction the road topology via multi-stage schemes according to various information, such as simple interaction [15], 3D road surface model [1], pre-defined classifiers [2] or aperiodic directional structure measurement [16]. However, when facing various road maps, these methods require time-consuming processes to select proper parameters.

To overcome the manual adjusting trouble for various situations, learning-based approaches have been widely used in road extraction. Multi-level networks [12] and higher-order CRF [17] are used to label road pixels by training models from massive road region samples. Recently, deep learning has developed rapidly, and it can be effectively applied to many fields, such as signal processing [18], agriculture [19], chemistry [20], medicine [21], etc. A recent road extraction methods, cascaded convolutional neural networks (CasNet) [11], achieves good results by constructing a unified network to extract road region maps and road centerlines.

These road extraction methods devoted to constructing end-to-end road extraction frameworks somehow lead to incomplete results, especially facing various road spectral conditions [22]. To get complete road topological networks, the other simple but effective way is to refine the extracted imperfect road topology, where few studies have concentrated [8,22]. Road structure refined CNN (RSRCNN) [22] is the only deep-learning-based work on road structure refinement, but, due to the absence of a unified consideration of the topological and spectral information, the extraction results are not satisfactory in some situations.

In this paper, we propose Multi-conditional Generative Adversarial Network (McGAN), a topology refinement network to repair the incomplete road networks. By utilizing existing road extraction methods to get an initial road map, the proposed McGAN is able to refine road maps to get complete topology structure. Specifically, the proposed McGAN includes two discriminators and a generator. One discriminator assists in reconstructing the road network instructed by spectral structures, while the other discriminator aims to obtain complete road networks based on the connectivity of initial topology. Then, the generator is trained jointly by these two discriminators. Such a structure makes the generator able to receive both the spectral and the topological information of the road region. The experiments demonstrated McGAN can produce a complete road network topology.

## 2. Related Work

According to previous surveys [14,23] and the latest road extraction works [2,15], road network extraction works can be approximately classified into three different types: feature-oriented, topology-oriented, and learning-based.

Early studies extract the roads based on the optical and geometric features, and many filters and line segment extraction schemes are designed, such as Kalman filters [24], directional filters [23,25], Gibbs point [26], line segments matching [27], etc. Peng et al. [28] proposed a multi-scale statistical data model to integrate the results of coarse resolution and fine resolution, as well as an outdated GIS map as the prior knowledge, but the results may contain many false detections and leave out the smaller roads. Based on orientation-based segmentation, Poullis and You [29] utilized a unified framework with Gabor filtering, tensor voting and segmentation to classify and segment the road area, but it performs poorly in cases where the color distributions of the background and foreground objects are very similar. Grote et al. [30] combined the radiometric and geometric features and extracted roads by building a subgraph to connect the possible road elements; however, the completeness drops sharply when facing trees or building shadows. Zhang et al. [31] utilized a semi-automatic road tracker to detect the road area dynamically, but it cannot continue past the abrupt appearance of large geometric and radiometric changes. Sghaier et al. [32] proposed a two-stage method based on road edge selection and the beamlet transformation to reconstruct the road network, which does not account for geometric and radiometric variability and the influence of occlusions. As mentioned above, these methods achieve relatively low accuracy, are limited to road networks with regular structures and are greatly affected by occlusions [3], thus they might fail for complex road conditions.

To address this problem, most recent road extraction methods contain a topology reconstruction scheme, aiming to produce a more complete road network with less isolate false fragments. Steger et al. [33,34] first used graph methods to reconstruct road network topology which is limited to lines with a certain range of widths. Peteri and Ranchin [35] extracted road area by defining the active contours under the graph system, they do not precisely matched the road centerlines.

Ziems et al. [1] integrated several models of different methods and analyzed these road extraction results for various remote sensing images. Unsalan et al. [15] proposed a robust automatic system containing road topology extraction, area detection, and graph-based reconstruction. Zang et al. [3] proposed an aperiodic directional structure measurement (ADSM) to extract road networks. Shi et al. [2] utilized a spectral and spatial classifier for road topology reconstruction, which can obtain an integrated network for regular roads, fails for complicated road junctions. Zang et al. [16] proposed a task-oriented enhancing technique for extracting road networks from satellite images, which is able to smooth high contrast textures and improve the performance of road feature extraction and topology reconstruction. However, to adapt to various road types, most of these works follow an ad-hoc multistage scheme, thus leading to the adjustment of multiple parameters and making it hard to apply in real-world datasets.

To avoid the manual operation process, learning-based methods have been paid much more attention. Early works [36,37] implement the prediction based on the features of a local context. Mnih and Hinton [12] first proposed the deep neural framework for road extraction and presented a spatial coherence based post-processing scheme to refine the road topology. However, it is restricted to the small context to label pixels. Yuan et al. [38] proposed a locally excitatory globally inhibitory oscillator network (LEGION) to group the well-aligned points, and further represent the roads based on these classified points. However, the results might have small broken lines. Wegner et al. [17] trained a higher-order CRF model and labeled the road area with thin chains of superpixels. For narrow roads, it might lose its effectiveness and cut them into small pieces. With the development of the convolutional neural network (CNN) [39], recent learning-based road extraction approaches either develop based on CNNs or the derived networks, such as FCN [40], ResNet [41], etc. Saito et al. [42] built a new system for multiple kinds of objects extraction, e.g. roads and buildings, based on a CNN-based extractor and classifier. Zhong et al. [43] analyzed many factors that may affect the roads and buildings extraction but left out the geometric feature of road topologies. Cheng et al. [11] proposed a novel network (CasNet) to unify the road region and topology extraction in a cascaded structure, and demonstrated the effectiveness on a new road segmentation and centerline dataset. A recent work [22] proposes a road structure refined CNN (RSRCNN), that considers not only the spatial information but also the geometric distribution, based on a novel road-structure-based loss function. Despite the huge promotion beyond previous methods, these methods still often present unsatisfactory results.

## 3. Topology Refinement via McGAN

Specific to the unsatisfactory extracted road networks, our motivation is to design a topology refinement framework to produce more complete road network. With the initial road network generated by the method in [3] (other road network extraction methods such as those in [10,15,44] are also acceptable), by combining the original image and the reference, McGAN is proposed to reconstruct and refine the road network topology. In the following sections, the architecture and loss functions of the proposed network are introduced in detail.

### 3.1. Network Architecture

The proposed McGAN is essentially a multi-conditional generative adversarial network. The input of the network contains the original image, an initial road network and a reference (ground truth). The whole network includes mainly two parts: First, it aims to employ the original spectral information to instruct the reconstruction. Second, the initial road network is considered as the other condition to refine the road network topology.

**Figure 2:**
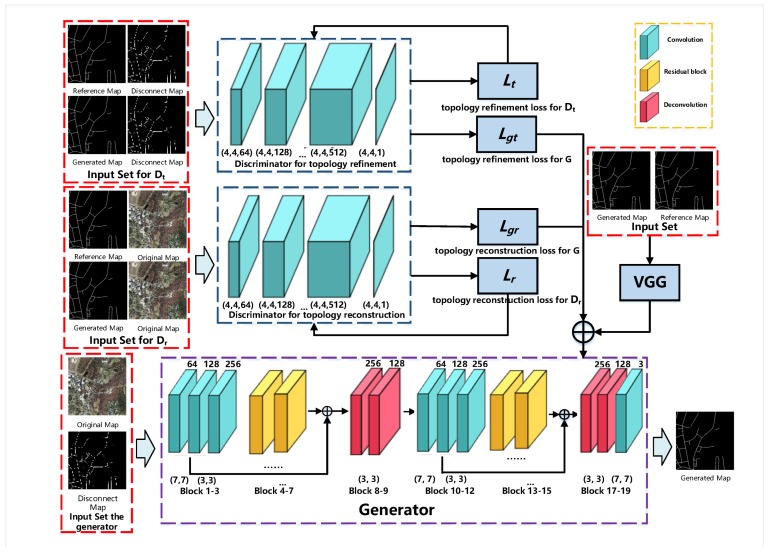
Network Architecture.

The architecture of the network is shown in Figure 2, where one generator, two discriminators and a VGG [45] branch are involved. One of the discriminators, denoted as $D_{r}$, focuses on the spectral performance of road area, which is trained by the reference, the original image and the output of the generator. The other discriminator, denoted as $D_{t}$, aims to refine the road network topology, and is trained by the reference, the initial road network and the output of the generator. The VGG branch performs as a feature-based extractor and comparator. Then, the output losses of $D_{r}$ and $D_{t}$ are combined with the VGG loss to jointly train the generator. The discriminator $D_{r}$ includes four two-dimensional convolutional layers. In general, the output of a discriminator is one bit, while, inspired by the previous work [46], the Markov random field is designed for the patch output, which means the discriminator works on a N×N patch. The scheme considers the spatial relationship of a certain pixel and its neighbors, which is more reasonable to calculate the output loss. Following this idea, through the experiments, the size of the patch is set as 70×70. For the discriminator $D_{t}$, the same structures are applied, and the only difference is that $D_{t}$ is trained by the reference and the initial road network. The generator is composed of an encoder and a decoder. The encoder consists of four residual blocks, three convolutional layers, and two deconvolutional layers. Then, the following decoder contains the symmetric structure as the encoding part. Inspired by Unet [47], we also add two skipped connections between the encoder and decoder to reserve the low-level features. Additionally, we also add a pre-trained VGG network to capture the structure features, thus making it better to compare the road network topology. Then, the generator is jointly trained by the losses of $D_{r}$, $D_{t}$ and VGG. Details of the discriminator and generator are shown in Table 1 and Table 2.

### 3.2. Network Loss Functions

According to the architecture of the network, three types of losses are involved. For the discriminator $D_{r}$, the corresponding loss $L_{r}$ can be written as:

(1) $$L_{r}\left(G,D_{r}\right)=E_{o,y\in P_{d}\left(o,y\right)}\left[logD_{r}\left(o,y\right)\right]+E_{o,x\in P_{d}\left(o,x\right)}\left[log\left(1-D_{r}\left(o,G\left(o,x\right)\right)\right)\right]$$

(2) $$L_{gr}\left(G\right)=E_{o,x\in P_{d}\left(o,x\right)}\left[log\left(1-D_{r}\left(o,G\left(o,x\right)\right)\right)\right]$$

where *o*, *x* and *y* represent the original image, the initial road networks and ground truth, respectively; G(·,·) represents the output of the generator; $D_{r}\left(\cdot \right)$ represents the output of the discriminator; and $P_{d}$ represents the distribution of the data. $D_{r}$ is trained under the adversarial scheme: the generator tries to output fake images that are as real as possible, while the discriminator tries to make the correct decision; such a training strategy can make the generator much stronger to produce desirable results. This loss function is designed to instruct the topology reconstruction based on original spectral information.

Then, to make the generator capable of obtaining the complete topology of road networks, another discriminator $D_{t}$ is designed to refine the potential incomplete structures of the initial road network. Specifically, the loss function of $D_{t}$ can be written as:

(3) $$L_{t}\left(G,D_{t}\right)=E_{x,y\in P_{d}\left(x,y\right)}\left[logD_{t}\left(x,y\right)\right]+E_{o,x\in P_{d}\left(o,x\right)}\left[log\left(1-D_{t}\left(x,G\left(o,x\right)\right)\right)\right]$$

(4) $$L_{gt}\left(G\right)=E_{o,x\in P_{d}\left(o,x\right)}\left[log\left(1-D_{t}\left(x,G\left(o,x\right)\right)\right)\right]$$

Guided by the original images, the loss $L_{t}$ is able to make the network learn how to connect the road fragment based on the initial road network, thus forming more complete road network topology.

To make the generator, denoted as *G*, capable of generating road networks with complete topology, the loss of *G* consists of two terms. The first term, denoted as $L_{g}$, is derived from the loss functions of the discriminators:

(5) $$L_{g}\left(G\right)=L_{gr}\left(G\right)+L_{gt}\left(G\right)$$

In addition, we also add a pre-trained VGG as another term to capture the fine structure features. Specifically, the pixel-wise differences between the feature maps extracted by VGG are summed under the L1 norm:

(6) $$L_{VGG}\left(G\right)=\sum_{k=i_{1}}^{i_{m}}\lambda_{k}{∥H_{k}\left(G\left(o,x\right)\right)-H_{k}\left(y\right)∥}_{1}$$

where $H_{k}$ denotes the *k*th layer of the pre-trained VGG network, $\lambda_{k}$ denotes the weight of the *k*th layer and $i_{1}∼i_{m}$ denotes the *m* extracted layers. The term $L_{VGG}\left(G\right)$ is designed due to the powerful ability of VGG to extract features, thus making it better to perceive the road network topology. Then, the generator is trained by the loss $L_{G}$:

(7) $$L_{G}\left(G\right)=L_{g}\left(G\right)+L_{VGG}\left(G\right)$$

With the designed losses above, the total objective of the network can be written as:

(8) $$G^{*}=arg\min_{G}\max_{D_{r},D_{t}}L_{total}$$

where $L_{total}$ is:

(9) $$L_{total}=L_{r}\left(G,D_{r}\right)+L_{VGG}\left(G\right)+\lambda_{t}L_{t}\left(G,D_{t}\right)$$

The aim of the scheme is to minimize the term of $L_{G}\left(G\right)$ to make the output of generator as similar as possible to the reference, and maximize the terms $L_{r}\left(G,D_{r}\right)$ and $L_{t}\left(G,D_{t}\right)$ to make the discriminators capable of identifying the fake results.

## 4. Results and Analysis

The proposed McGAN contains mainly three loss functions: $L_{r}$, $L_{t}$ and $L_{VGG}$. Specifically, the $L_{r}$ loss is employed to generate the road topology. Then, to capture the fine features and locate the potential incomplete topology structures, the VGG loss is adopted to train the generator. Finally, to further confirm the alternative structures and teach the generator to refine the road network topology, the $L_{t}$ loss is adopted.

In this section, to demonstrate the validation of the topology refinement and to evaluate how different parts of the losses affect the results, we first create several groups of experiments to demonstrate the validity of our method (Section 4.2 and Section 4.3). Meanwhile, we also show the performance of McGAN on the three datasets (Section 4.3). Finally, our approach is compared with several state-of-the-art approaches (Section 4.4).

### 4.1. Implementation Details

***Datasets.*** In the experiment, three datasets are selected to comprehensively evaluate the proposed approach. The first two are publicly released by previous works [11,48], along with the accurate reference as the training label. The other one is from the work in [16] provided by the author.

The first dataset, Google Earth dataset, is public, released by Cheng et al. [11] and can be downloaded at the address (http://www.escience.cn/people/guangliangcheng/Datasets.html). The dataset, composed of 224 very high resolution (VHR) urban road images from Google Earth and including 180 training samples and 44 testing samples, also provides the largest accurate segmentation maps and centerline maps. The second dataset we used for comparison is Massachusetts Roads dataset, which was released by Mnih et al. [48] and can be downloaded at the address (http://www.cs.toronto.edu/~vmnih/data/). The resolution of the image is 1 m with the size of 1500 × 1500 pixels, which covers more than 2600 km^2^ in total and includes 1108 training samples and 63 testing samples. The third dataset is the remote sensing images with resolution 0.5 m, which were captured by Pleiades-1A remote sensing satellite and cover the entire Shaoshan City (Hunan province, China). The reference was obtained by ground survey and provided by China Transportation & Telecommunication Center. Shaoshan City covers 247 km^2^ in the mid-south of China, where most area is mountainous or rural. The size of the whole image is 28,648 × 37,929 pixels, which was then divided into 1000×1000 patches, and we evaluated our approach on each patch and finally merged them together.

The first two datasets are suitable for deep-learning based road extraction methods, and the data can be used directly. For all three datasets, to demonstrate the valididty of the topology refinement, the initial road networks were selected as a traditional method in [3] or a modified conditional GAN.

***Implement Details.*** PyTorch framework was applied to build the entire network on a PC with one Titan X GPU. The training process was based on Adam solver [49] and the learning rate was 0.001. The weights of network were initialized from a Gaussian distribution with mean μ=0 and standard deviation σ=0.02. The number of training epochs was set as 150. For the parameters of the network, three layers of the VGG was extracted to calculate the $L_{VGG}$ loss, namely the 7th, 12th and 15th layers, and the corresponding λ was set as 1. The $\lambda_{t}$ of the weight of $L_{t}$ was 0.8.

***Quantitative Measurements.*** The quantitative evaluation measurements were selected as the commonly used standards provided by previous works [50,51]: recall, precision and F1 score. They can be written as:

(10) $$\begin{array}{c} \mathrm{recall}\left(R\right)=\frac{TP}{TP+FN}\\ \mathrm{precision}\left(P\right)=\frac{TP}{TP+FP}\\ F1\;\mathrm{score}\left(F\right)=\frac{2\cdot TP}{2\cdot TP+FN+FP} \end{array}$$

where TP, FN, and FP denote true positive, false negative, and false positive, respectively.

### 4.2. Evaluation of the Network Performance

**Figure 3:**
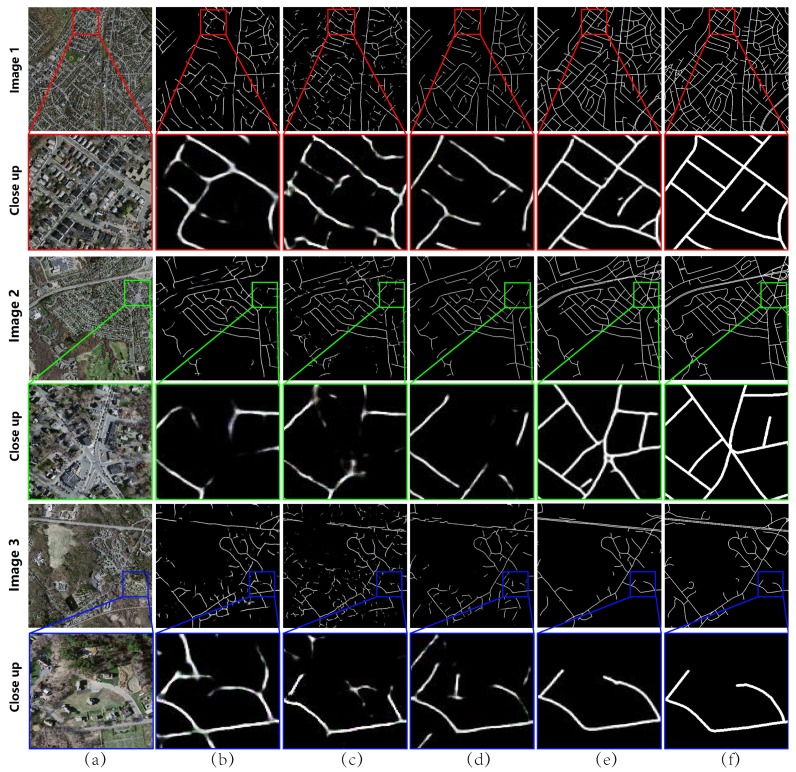
Typical comparison on parameters: (**a**) original image; (**b**) result of the method in [3] (the initial road network); (**c**) result without $L_{VGG}$; (**d**) result without $L_{r}$; (**e**) result of McGAN; and (**f**) result of the reference map. The red boxes in (**b**–**d**) indicate some poor extraction parts compared with (**e**).

A group of experiments was designed to evaluate how the loss functions of $L_{r}$ and $L_{VGG}$ affect the results. In Figure 3, Column (a) is the input image, Column (b) is the result of the method in [3], Column (c) is the result without the $L_{VGG}$, Column (d) is the result without $L_{r}$ loss, Column (e) is the result of the integrated network with the initial roads extracted by previous work [3], and Column (f) shows the ground truth. From the results, it was found that the method in [3] lost some salient road structures, thus leading to various “gaps” in the road networks, as highlighted in the red box of Column (b). Without $L_{VGG}$, isolated road fragments were observed since the fine structures could not be captured, as highlighted in the red box of Column (c). Without the $L_{r}$ loss, some redundant connections or gaps appeared due to the lack of spectral information and the network may fail to correctly connect the fractured topology, as highlighted in the red box of Column (d). While for the proposed approach, the refined road network in Column (e) is much more complete and correct compared with the initial network (b), even when the input images were very challenging for road network extraction.

### 4.3. Evaluation on Various Datasets

We evaluated how the loss $L_{t}$ affected the results. Notice that when, we kept the $L_{r}$ and $L_{VGG}$ and removed $L_{t}$, the network turned into a Single-conditional GAN (ScGAN), which extracted the road network in an end-to-end manner. We also showed the performance of McGAN on three datasets. The first two, with the resolution of 1 m and 1.2 m, are released by the previous works [11,48], where plenty of rural region roads are involved and the corresponding reference is provided by the datasets. The other dataset, with the resolution of 0.5 m, was captured by Pleiades-1A satellite and the reference is provided by China Transportation & Telecommunication Center.

**Figure 4:**
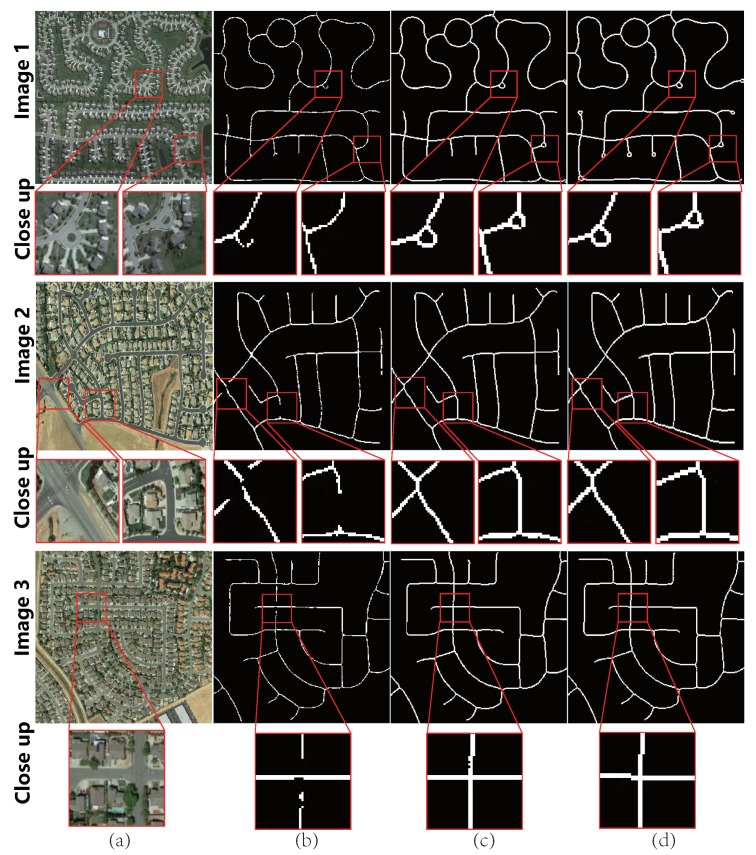
Visualized results of typical examples on Google Earth dataset: (**a**) original image; (**b**) result of ScGAN (without $L_{t}$); (**c**) result of McGAN; and (**d**) ground truth.

***Test on Google Earth dataset.*** The testing results on Google Earth dataset are shown in Figure 4. In Column (a), the spectral performance of road region is not that salient, thus leading to unsatisfactory initial extraction results without $L_{t}$, as shown in Column (b). The refinement result is shown in Column (c). It was found that, even though there were many interferences such as buildings or occlusions, by considering the spectral and topological structures, McGAN could obtain more complete topology based on (b). The average quantitative measurements over the whole data are listed in the second row of Table 3.

**Figure 5:**
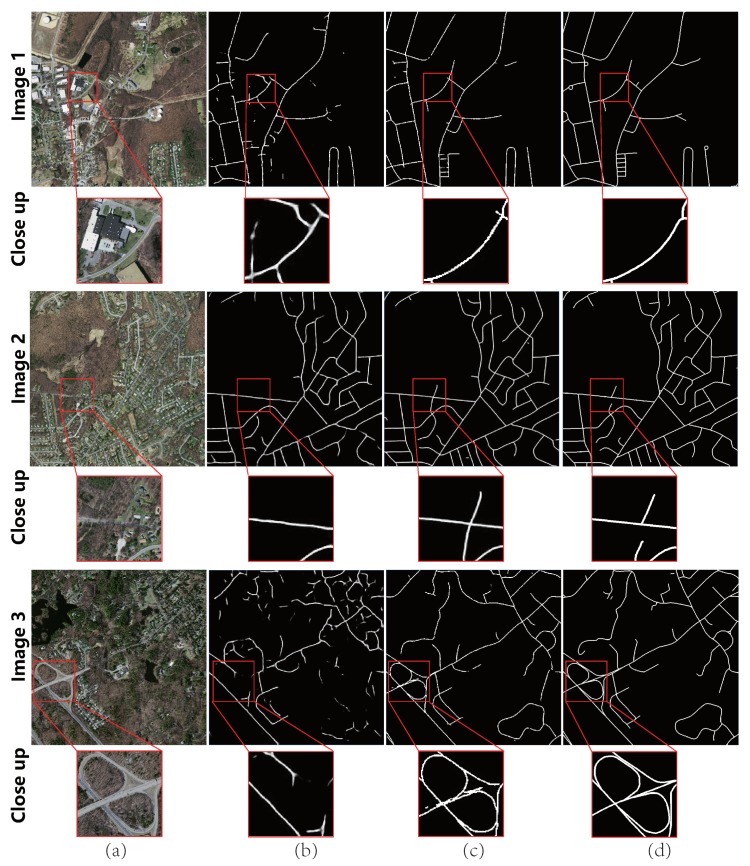
Visualized results of typical examples on Massachusetts dataset: (**a**) original image; (**b**) result of ScGAN; (**c**) result of McGAN; and (**d**) ground truth.

**Figure 6:**
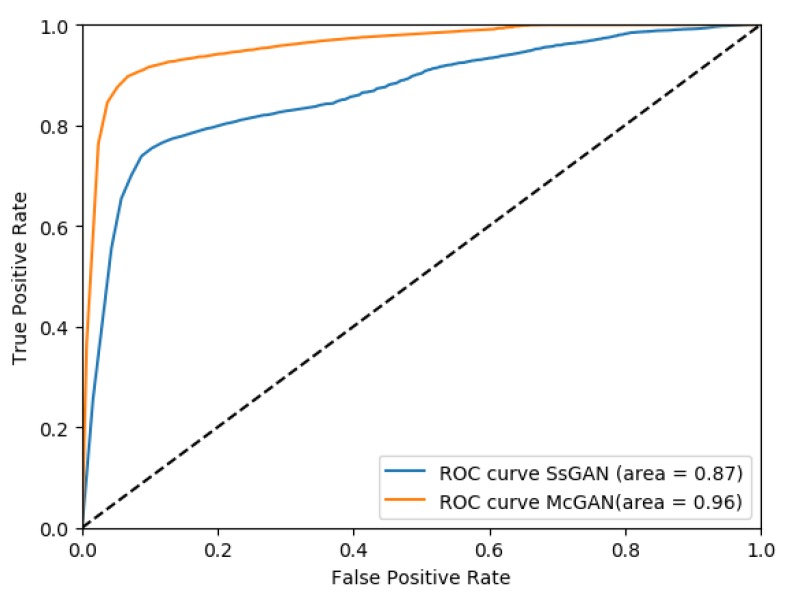
ROC curve of Image 3 in Figure 5.

***Test on Massachusetts Roads Dataset.*** Then, the proposed approach was tested on the Massachusetts Roads Dataset. Similar to the last step, the ScGAN was applied to generate the initial road networks. Selected examples are shown in Figure 5, Column (a), and the corresponding initial road networks are shown in Column (b). According to the results, some of the terrain boundaries were misidentified as roads, thus leading to some isolated road fragments. For our result, these road fragments were well removed and the overall *F1 score* achieved almost 85%. The average quantitative measurements of this dataset are listed in the third row of Table 3. To better show the performance of our method, we also made a Receiver Operating Characteristic (ROC) curve of Image 3 as an example, as shown in Figure 6. For each pixel in the image, we calculated its shortest distance to the true road centerline, which is opposite to the probability of belonging to the road. It can been seen that McGAN had higher precision than ScGAN.

**Figure 7:**
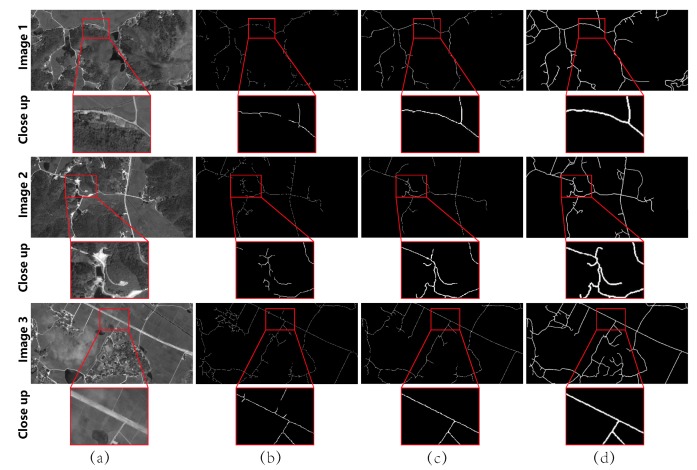
Visualized results of typical examples on Shaoshan dataset: (**a**) original image; (**b**) result of the ScGAN; (**c**) result of McGAN; and (**d**) ground truth.

***Test on Shaoshan dataset.*** For this dataset, we performed tests on the image of the whole Shaoshan City. The selected example, as shown in Figure 7, is a typical case for the rural region, where the spectral performance of the roads is rather variable. The initial road networks, extracted by ScGAN, suffered from incomplete topology in many places due to the curve roads, shadows and occlusion, as highlighted in the red box in Column (b). For McGAN, most of these cases were well handled, as shown in Column (c). The average quantitative measurements over the whole image are listed in the fourth row of Table 3.

### 4.4. Comparisons with State-of-the-Art Approaches

Some of the latest road network extraction or topology reconstruction approaches were employed for comparison. The selected baseline dataset is public by Mnih [48], and is rather challenging for road network extraction, where the curved roads, interference of buildings, shadows and occlusion are often observed, thus making it very close to the real case in practice. Based on these data, several latest approaches, including RSRCNN [22] and CasNet [11], were employed for evaluation.

**Figure 8:**
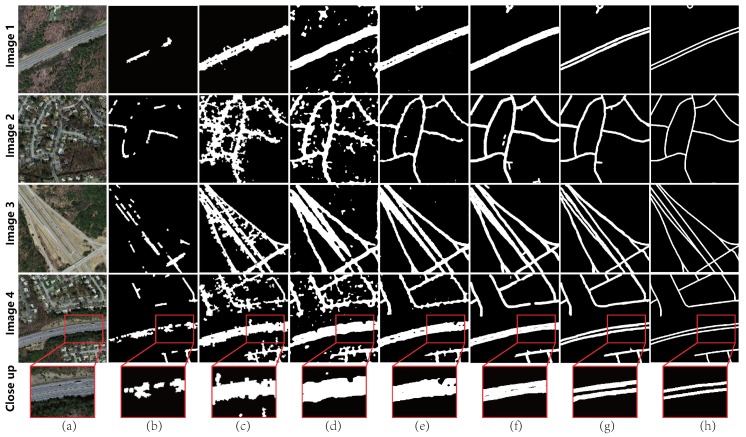
Comparison with state-of-the-art methods (the results in [17,22,43,52] were provided by Wei et al. [22]): (**a**) original image; (**b**) result of [17]; (**c**) result of [52]; (**d**) result of [43]; (**e**) result of [22]; (**f**) result of [11]; (**g**) result of McGAN; and (**h**) ground truth.

In the comparison, the same patches applied in previous work [22] were considered, and the corresponding results are shown in Figure 8, where Column (a) is the input image; Column (b)–(g) are the results corresponding to methods in [11,17,22,43,52] and our approach, respectively; and Column (h) is the ground truth. The results of previous work [17,22,43,52] were provided by [22], and the results of [11] were implemented with few changes to adapt to the dataset.

From the results, it was found that that, for the presented challenging cases, feature-based CRF scheme [17,52] performed poorly due to the interference of terrain or buildings, and the results suffered either incomplete topology or heavy false alarm. Learning-based algorithms [11,22,43] had better performance. For the result of the method in [43], major road network topology structures were captured, but errors often occurred around the buildings. The approaches in [11,22] are derived from CNNs, which could produce high-quality extraction results. However, some “gaps” were still observed at the road region with shadows or occlusion, and fine structures failed to be identified, such as the roads marked with double lines. Utilizing the method in [11] to extract initial topology, our approach could provide the road network with more complete topology, as shown in Column (g).

Corresponding statistics are shown in Table 4. It was found that the employed dataset is rather challenging for the road network extraction, and previous approaches [17,22,43,52] performed unsatisfactory, where either the recall was lower than 0.6 or the precision was lower than 0.75 (a previous road network extraction work [53] recommends values of 0.6 and 0.75 for recall and precision, respectively, as the baselines for practice). The approach in [11] performed well for this dataset and apparent improvement was observed for the overall F1 score. McGAN performed quite well, where the recall improved by more than 3%, and a 7% improvement for the precision was also observed.

## 5. Conclusions

This paper proposes a novel multi-conditional generative adversarial network (McGAN) for the road topology refinement. McGAN consists of two discriminators and a generator. The two discriminators are aimed for the reconstruction and refinement of road topology, and the generator is trained jointly by them along with a VGG network. McGAN can take both the topological and the spectral feature into account and obtain more complete road network topology compared with previous works. In fact, the Multi-conditional framework is not restricted to road extractionl for example, for the 3D Lidar point clouds processing, if we consider the 2D and 3D information together, it may produce better results. The addition of road region maps can promote the accuracy of road network extraction; however, it also increases the labeling burden when preparing the training data, which is very tedious and time consuming (fortunately, there are two public datasets). For some extreme situations, McGAN also failed to refine the incomplete structures; the reason may be that we only extract high-level features by the two discriminators, which may miss some fine-grained parts. Further research can attempt to utilize low-level spectral and topological features, such as the slope, curvature or connectivity, for better fusing of the instruction of road region and topology into a whole network.

## Figures and Tables

**Table 1:** Generator architecture.

Name	Network Structure
Block 1	Conv(7, 7, 64), stride = 1; InstanceNorm
Block 2	Conv(3, 3, 128), stride = 2; InstanceNorm; ReLU
Block 3	Conv(3, 3, 256), stride = 2; InstanceNorm; ReLU
Block 4	Residual block
Block 5	Residual block
Block 6	Residual block
Block 7	Residual block; Connect to Block 3
Block 8	DConv(3, 3, 256), stride = 3; InstanceNorm; ReLU
Block 9	DConv(3, 3, 128), stride = 3; InstanceNorm; ReLU
Block 10	Conv(7, 7, 64), stride = 1; InstanceNorm
Block 11	Conv(3, 3, 128), stride = 2; InstanceNorm; ReLU
Block 12	Conv(3, 3, 256), stride = 2; InstanceNorm; ReLU
Block 13	Residual block
Block 14	Residual block
Block 15	Residual block
Block 16	Residual block; Connect to Block 12
Block 17	DConv(3, 3, 256), stride = 2; InstanceNorm; ReLU
Block 18	DConv(3, 3, 128), stride = 2; InstanceNorm; ReLU
Block 19	Conv(7, 7, 3), stride = 1; Tanh

**Table 2:** Discriminators architecture.

Name	Network Structure
Block 1	Conv(4, 4, 64), stride = 2; LReLU
Block 2	Conv(4, 4, 128), stride = 2; InstanceNorm; LReLU
Block 3	Conv(4, 4, 256), stride = 2; InstanceNorm; LReLU
Block 4	Conv(4, 4, 512), stride = 1; InstanceNorm; LReLU
Block 5	Conv(4, 4, 1), stride = 1

**Table 3:** Quantitative statistics of McGAN on all the three datasets.

	Measurement	Recall	Precision	F1 Score
Data				
Google Earth	Result of ScGAN	0.895	0.914	0.904
	Result of McGAN	0.953	0.961	0.957
Massachusetts	Result of ScGAN	0.816	0.794	0.805
	Result of McGAN	0.858	0.841	0.849
Pleiades-1A	Result of ScGAN	0.801	0.783	0.792
	Result of McGAN	0.842	0.839	0.841

**Table 4:** Quantitative statistics of different methods on Massachusetts Dataset.

Method	Recall	Precision	F1 Score
Wegner et al. [17]	0.322	0.405	0.359
Wegner et al. [52]	0.679	0.471	0.556
Zhong et al. [43]	0.686	0.435	0.532
Wei et al. [22]	0.729	0.606	0.662
Cheng et al. [11]	0.783	0.812	0.797
McGAN	0.858	0.841	0.849
